# Association Between Interleukin-6 -572 C>G and -174 G>C Polymorphisms and Hypertension: A Meta-analysis of Case-control Studies

**DOI:** 10.1097/MD.0000000000002416

**Published:** 2016-01-15

**Authors:** He Ma, Guixiang Sun, Wei Wang, Yunti Zhou, Dang Liu, Yue Tong, Zhaojun Lu

**Affiliations:** From the Department of Public Health, Xuzhou Medical College, Xuzhou, Jiangsu, China (HM, GS, WW, YT, ZL); and Department of General Practice, Xuzhou Medical College, Xuzhou, Jiangsu, China (YZ, DL).

## Abstract

Whether hypertension is associated with −572 C>G or −174 G>C polymorphism in interleukin (IL)-6 genes still remains hazy and ambiguous.

We conducted a meta-analysis to offer a more reliable and clearer evaluation about the association.

Electronic literature databases including PubMed, Web of Science, EMBASE, Google Scholar, Chinese National Knowledge Infrastructure and Wanfang database were searched.

The study included the following: evaluating associations between −572 C>G or −174 G>C polymorphism in IL-6 gene and hypertension; case-control design; essential information must be offered; precise diagnostic criteria of hypertension; and no language restriction.

Patients who met the diagnostic criteria and controls without a history of hypertension were included. Interventions were not available.

A quality assessment was conducted using Newcastle-Ottawa scale. Combined odds ratios with 95% confidence intervals were calculated in 5 genetic models. Sources of heterogeneity were explored by subgroup analysis, meta-regression, and Galbraith plots. Finally, test for publication bias was performed to prove the stabilization.

Fifteen studies were finally included. Eleven articles were judged high-quality reports. Overall, the −572 C>G polymorphism was proved to be significantly associated with hypertension in 4 genetic models. Subgroup analysis based on ethnicity revealed significant associations in Asian population in recessive model and homozygote comparison. The association in Europeans and Mid-East required further confirmation. No significant association was observed between the −174 G>C polymorphism and hypertension under all of the genetic models.

The limitations of the study were the following: restrictive number of eligible studies limited the extrapolation range in subgroup analysis; gene–environment factors could not be described due to lack of data; some relevant studies could not be included because of various reasons.

Current researches supported the association between the development of hypertension and the −572 C>G rather than −174 G>C polymorphism. Future well designed epidemiological studies may evaluate the possible gene–environment interactions.

## Abstract 2

Supplemental Digital Content is available in the text

## INTRODUCTION

Hypertension is considered one of the leading causes of human death and disability.^1,2^ According to the epidemiologic data, approximately 1.56 billion people have hypertension or related symptoms, which is 29% of the adult population in the world.^3^ These problems are aggravated by the aging of population worldwide. Additionally, a significant proportion of patients have persistent blood pressure elevation called “resistant hypertension,” which rarely responds to antihypertensive drugs. Few treatments, such as renal sympathetic denervation, have turned out to be effective.^4^ Hypertension can also be a risk factor for stroke, myocardial infarction, end-stage renal disease and other serious illnesses.^5,6^ Therefore, priority should be given to understand the pathogenesis of hypertension and controlling this trend. Hypertension is not a simple entity, but a complex and multifactorial trait, which is usually associated with different combinations of genes, demographic factors, environmental elements, and additional cause, such as primary hyperaldosteronism, obstructive sleep apnea, and chronic renal disease.^4^ Despite the well recognized environmental or demographic factors, there is a consensus that a correlation exists between hypertension and gene mutations, and 150 genes are suspected to be related to hypertension.^7^

Interleukin (IL)-6 is a 23.7-kDa pleiotropic cytokine, secreted by cells from the immune system, cardiovascular components, and adipose tissue.^8^ It mainly functions in inflammation progression and is regarded as an endogenous pyrogen that induces fever in patients with infection. Moreover, IL-6 has a wide variety of biological functions. Lots of studies have shown that IL-6 levels are positively correlated with blood pressure,^9,10^ and its genetic polymorphism may have a connection with hypertension.^11,12^ The gene encoding IL-6 is located at chromosome 7p21 and contains 6 exons with a 1.3-kb coding sequence (size 6119 base pairs). Most studies have focused on the promoter region of the IL-6 gene because many polymorphisms are identified in this region, including −598A/G, −597G/A, −572 C>G, and −174 G>C. Of these polymorphisms, the 2 most commonly involved and researched are −572 C>G and −174 G>C. The polymorphism −572 C>G, known as −634 C>G or 1800796, is at base pair 572. Another polymorphism, −174 G>C, which is designated as rs1800795 as well, is a G-to-C transition located in the promoter 174 bp upstream of the start site of transcription and is found to be in linkage disequilibrium with −597G>A. Both of the polymorphisms are reported to result in alterations of the transcription rate of IL-6,^13^ although their geographical and ethnic distributions are quite different.^14,15^

A considerable number of studies have been performed to figure out whether there is an association between hypertension and −572 C>G polymorphisms, but the results are equivocal.^16–18^ A similarly chaotic situation appears in the −174 G>C polymorphism as well. Wang et al^19^ suggested a correlation between −174 G>C and coronary artery disease, whereas Ghazouani et al^20^ and Tong et al^21^ did not find this correlation, although all 3 studies were aimed to assess coronary artery disease and not hypertension. Due to the limited study populations, the distributions of the polymorphism in other areas remain unknown. In light of this situation, meta-analyses have been performed to obtain a more convincing report by summarizing research results with enhanced precision. In 2009, Niu et al^22^ conducted a case-control study in Shanghai and a meta-analysis to investigate the relationship between the −174 G>C polymorphism and hypertension, and concluded that the C allele helps decrease the risk of developing hypertension. However, this study only included Chinese volunteers, and thus the results cannot be extrapolated to broader populations. Additionally, new articles have been recently published, and these articles should be fully merged and updated in the new meta-analysis. On the contrary, no meta-analysis addressing the −572 C>G polymorphism has ever been conducted. To accomplish this task and to offer a more reliable and clearer evaluation, we performed a meta-analysis of all eligible studies regarding the association between the −572 C>G or −174 G>C polymorphisms and hypertension.

## MATERIALS AND METHODS

The PRISMA protocol was prospectively performed.

### Search Strategy

Computer searches were performed by one investigator (HM) in electronic literature databases including PubMed, the Web of Science, EMBASE, the Chinese National Knowledge Infrastructure (CNKI), and the Wanfang database, to identify all relevant studies regarding the association between −572 C>G and −174 G>C polymorphisms in IL-6 and hypertension susceptibility. A supplemental search was also manually conducted in the references of the retrieved papers and “Similar Articles” from PubMed. The following detailed search items and key words were used: (“hypertension” or “high blood pressure” or “hypertensive”) and (“IL 6” or “Interleukin 6”) and (“polymorphism” or “mutation”). The Google Scholar literature database was also included in the search strategy using “(“hypertension” OR “blood pressure”) AND (“polymorphism” OR “mutation”) AND (“il 6” OR “interleukin 6”) AND (“172” OR “574” OR “634” OR “1800796” OR “1800795”) –pulmonary”. The last search was updated through January 9, 2015. Ethical approval was waived because this study is a meta-analysis.

### Inclusion and Exclusion Criteria

Two investigators (HM and GS) independently evaluated articles collected from databases and references according to the criteria below. A consensus was achieved in instance of disagreements between the 2 authors. EndNote X7 (Thomson Corporation, CT) was used to merge the retrieved citations.

Studies that fulfilled all the following criteria were enrolled in the meta-analysis: evaluations of the associations between −572 C>G or −174 G>C polymorphism in the IL-6 gene and hypertension; case-control design; sample size, genotype distributions, or other essential information required to estimate the odds ratios (ORs) and 95% confidence intervals (95% CIs) (when overlapping data were presented in more than 1 study, either the newest study or the study with the largest sample size was selected in our meta-analysis); healthy individuals or patients without a history of hypertension were included in the control groups; diagnostic criteria for hypertension was: systolic blood pressure above 140 mm Hg and/or diastolic blood pressure above 90 mm Hg^23^; and no language restriction.

Studies that met one of the following criteria were excluded: not relevant to hypertension, the −572 C>G polymorphism or the −174 G>C polymorphism; not designed as case-control studies; detailed and usable data, for example, the genotype frequency or number, were not provided for extraction; and case-only studies without control groups, case reports, reviews, comments, abstracts, editorials, or animal studies.

### Data Extraction

Two investigators (HM and GS) independently extracted the data and information from the included studies. A discussion took place to achieve consensus and the full texts were examined again when the investigator opinions diverged. We contacted the authors to request necessary data if some data were missing. Data extracted from the studies included the following: the first author's name, the publication year, the country, the ethnicity, the source of the control, the age, the sex, the genotyping method, the IL-6 polymorphism type, the hypertension type, the Hardy–Weinberg equilibrium (HWE) in the controls (*P* value), the covariates adjusted in the logistic regression in original studies, the sample size, and the count of each genotype in cases and controls.

### Quality Score Assessment

The Newcastle-Ottawa scale (NOS)^24^ was used by 2 investigators (WW and YZ) to independently assess the quality of the eligible studies. NOS evaluated studies with a star-rating system ranging from 0 (the lowest quality) to 9 (the highest quality) stars, which was based on 3 study components, including selection, comparability, and outcome assessment. Articles that were awarded 5 stars or more were considered high-quality studies, whereas the other studies were considered of low quality. Discrepancies between the 2 investigators were solved by discussions to re-evaluate the methodological quality.

### Statistical Analysis

The pooled ORs with 95% CIs were used to measure the strength of the associations between the 2 mutations and hypertension, using the Z-test. Values of *P* <0.05 were considered to be significant. Five different genetic comparison models were performed for the OR calculation (A is the minor allele): allelic comparison (A vs B), heterozygote comparison (AB vs BB), homozygote comparison (AA vs BB), dominant model (AA + AB vs BB), and recessive model (AA vs AB + BB). The degree of heterogeneity among studies was estimated by the inconsistency index (*I*^2^), ranging from 0% to 100%, together with a *P* value from Q-test. *I*^2^ ≤25% represented no heterogeneity; There was low heterogeneity when 25% < *I*^2^ ≤ 50%; if 50% < *I*^2^ ≤ 75%, the result was considered to have significant heterogeneity and *I*^2^ >75% indicated high heterogeneity. A random-effects model (DerSimonian–Laird method) was applied to merge the ORs and 95% CIs in all of the genetic models. Moreover, a supplementary analysis was added using a fixed-effects model (Mantel–Haenszel method) to achieve high test power and statistic efficiency, only if *I*^2^ ≤50%. Stratification analyses based on ethnicity and meta-regression were conducted to explore the source of heterogeneity when the results were found to have significant heterogeneity. The following characteristics were included as covariates in the meta-regression: type of disease (patients only having hypertension vs those having hypertension together with another disease), sex (only women vs only men vs both women and men), genotyping methods (polymerase chain reaction-restriction fragment length polymorphism [PCR-RFLP] vs not PCR-RFLP), and the source of the controls (population-based vs hospital-based). Moreover, Galbraith plots were conducted to further investigate heterogeneity.

Egger linear regression test and Begg test were utilized to assess the possibility of publication bias if the final number of eligible studies was above 10. When the number of studies was lower than 10, a modified Egger test, that is, Harbord method, was conducted instead of the conventional methods because it has a lower false-positive rate and better statistical power with relatively modest numbers of enrolled studies.^25^ Once publication bias was demonstrated to exist (*P* < 0.05), the “trim and fill” method was used as an adjustment. Additionally, to test the stability of the meta-analysis, a sensitivity analysis was conducted by excluding one study at a time and then calculating the pooled effect sizes of the remaining studies.

The HWE was calculated by using the chi-square test in every control group to ensure that the group represented normal and healthy people.

Excel 2013 (Microsoft Corporation, WA) was used for the data collection and the initial management. Meta-analyses were performed with Stata statistic software version 12.0 (Stata Corporation, College Station, TX).

## RESULTS

### Study Identification and Characteristics

**FIGURE 1:**
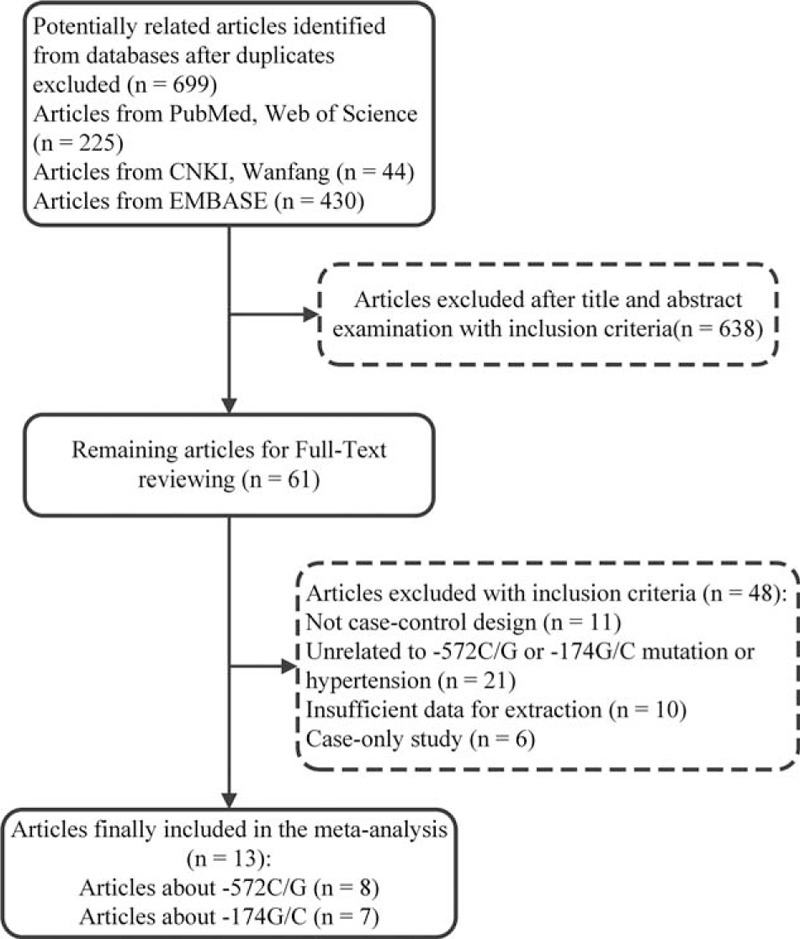
Flow chart of the selection process for including article.

The PRISMA checklist is presented in the Supplemental Content (Table S1). After removing duplicate, 699 articles were found to be relevant to the key words and search strategy by checking electronic databases and reference lists, including 104 articles from PubMed, 121 from the Web of Science, 430 from EMBASE, and 44 from the CNKI and Wanfang databases. During title and abstract examination, 638 articles met the exclusion criteria and were excluded. We removed 48 articles in the full-text review process. Among them, 11 papers did not have a case-control design^15,26–35^; 21 papers were unrelated to the main topic^9,20,36–54^; 10 papers did not have sufficient data for extraction^55–64^; and 6 papers were case-only studies without controls.^65–70^ From the Google scholar database, we retrieved 7400 articles at first. Then, we found that the additional studies were all replicates from the other databases after we removed the articles that met the exclusion criteria. Finally, 13 articles^11,12,16–18,71–78^ met all of the inclusion criteria and were enrolled in the meta-analysis, which contained 10 articles in English and 3 articles in Chinese (Figure 1), although language was not restricted in the search protocol or practice. Among them, 2 articles provided data regarding both of them; therefore, we considered each article as 2 studies. In total, 15 studies from 13 articles containing 1532 cases and 1374 controls about −572 C>G, in addition to 1231 cases and 1122 controls about −174 G>C, were included in the meta-analysis.

The main characteristics are listed in Tables 1 and 2. These studies were performed in China, Japan, British, Italy, German, Russia, Turkey, and Egypt, and were divided into 3 regions: “Asian”, “European,” and “Mid-East.” The control groups in all of the studies were population-based except in the study by “Babel et al”,^78^ which included hospital-based controls. Eight studies adopted PCR-RFLP for genotyping, whereas 7 studies used other genotyping methods. HWE was tested among all studies and no study was inconsistent, except for the study by Pola et al.^77^

**TABLE 1:**
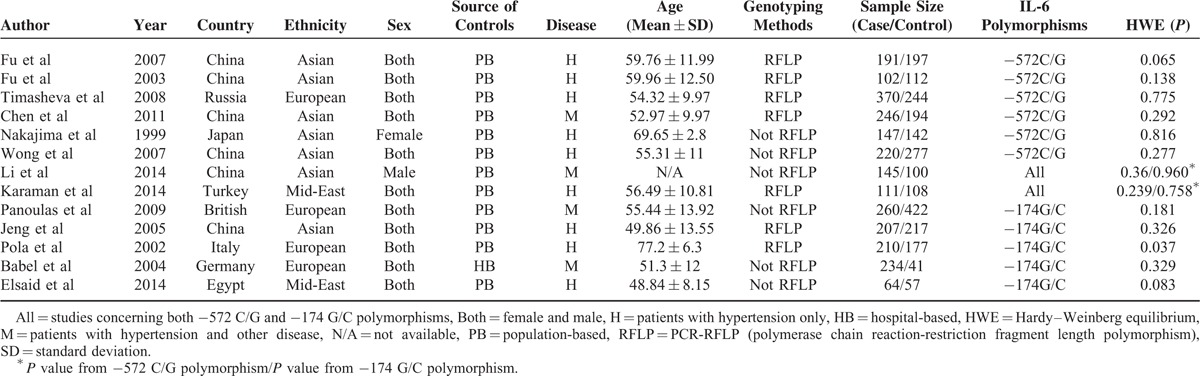
Baseline Characteristics of the Eligible Studies Included in the Meta-analysis

**TABLE 2:**
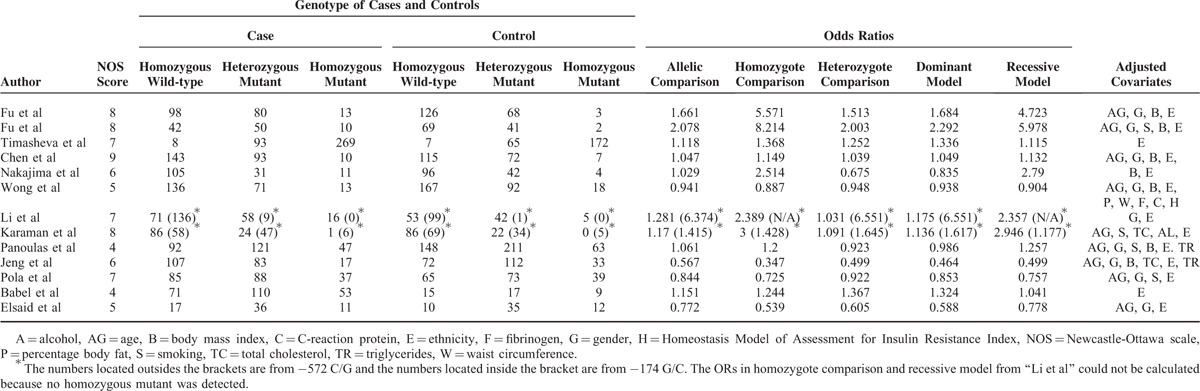
Quality Scores, Genotypes and Adjusted Covariates of the Eligible Studies

### Study Quality Assessment

The result of the NOS assessment is shown in the Supplemental Content (Table S2). According to the protocol, 11 articles were regarded as high-quality reports. One article earned the highest reward, with 9 stars, and 2 other articles scored the worst score, with only 4 stars. Two articles obtained the maximum 4 stars for study population selection. Four articles won full marks in the comparability component because they adjusted for age, sex, and other items. Ten articles were awarded the maximum of 4 stars based on the outcome assessment (−572 C>G).

### Statistical Analyses

**FIGURE 2:**
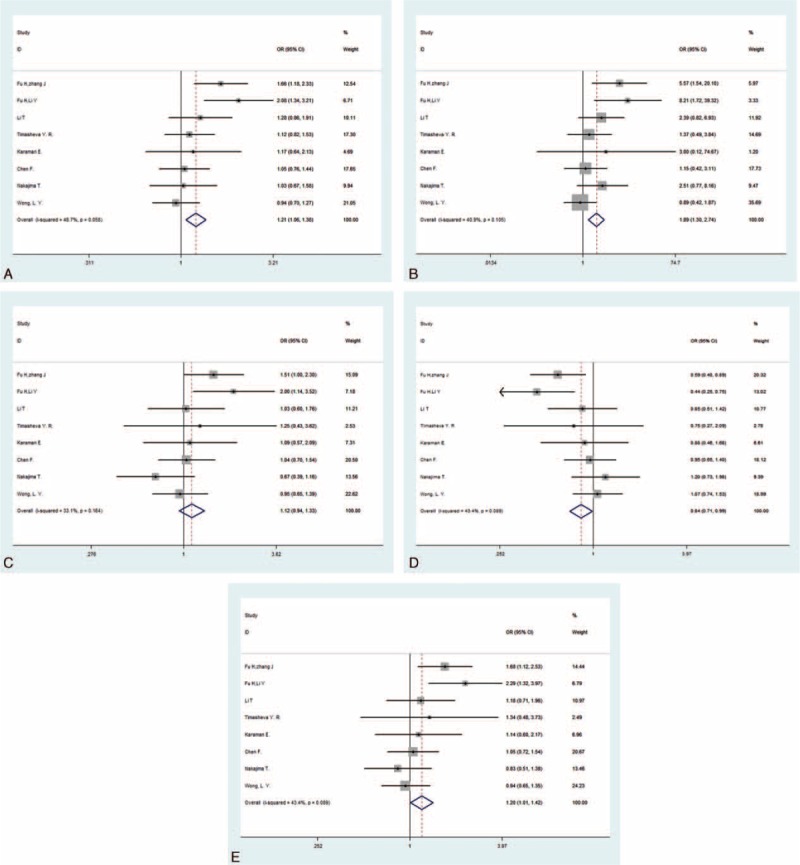
Forest plots for −572 C>G polymorphism in the overall analysis. The summary pooled ORs and 95% CIs are indicated by the white diamonds. (A) Allelic comparison (G vs C); (B) homozygote comparison (GG vs CC); (C) heterozygote comparison (CG vs CC); (D) dominant model (GG + CG vs CC); (E) recessive model (GG vs CG + CC).CI = confidence interval, OR = odds ratio.

No significant heterogeneity was detected in all of the 5 genetic models, leading to the application of the fixed-effects model (Table 3). There was a significant association between the −572 C>G polymorphism and hypertension in all of the models except the heterozygote comparison (G vs C: OR = 1.211, 95% CI = 1.064–1.378, *P* = 0.004; GG vs CC: OR = 1.886, 95% CI = 1.297–2.742, *P* = 0.001; CG vs CC: OR = 1.118, 95% CI = 0.939–1.332, *P* = 0.212; GG + CG vs CC: OR = 1.196, 95% CI = 1.011–1.415, *P* = 0.037; and GG vs CG + CC: OR = 1.427, 95% CI = 1.096–1.856, *P* = 0.008) (Figure 2). All of the genetic models indicated that the frequencies of the mutant allele G in patients with hypertension were higher than those in healthy controls. To ensure accuracy and stability, we also applied the random-effects model. The significance still existed in 3 genetic models (G vs C: OR = 1.230, 95% CI = 1.022–1.481, *P* = 0.029; GG vs CC: OR = 2.003, 95% CI = 1.167–3.439, *P* = 0.012; CG vs CC: OR = 1.123, 95% CI = 0.899–1.403, *P* = 0.306; GG + CG vs CC: OR = 1.215, 95% CI = 0.962–1.536, *P* = 0.102; and GG vs CG + CC: OR = 1.712, 95% CI = 1.077–2.723, *P* = 0.023, see Figure S1, Supplemental Content, which illustrates the pooled effect sizes of the −572 C>G polymorphism using the random-effects model). We performed a subgroup analysis to discover whether ethnicity could influence the frequency of the G allele and found that significance still existed in the “Asian” recessive model and homozygote comparison, but disappeared in the other models (Table 3).

**TABLE 3:**
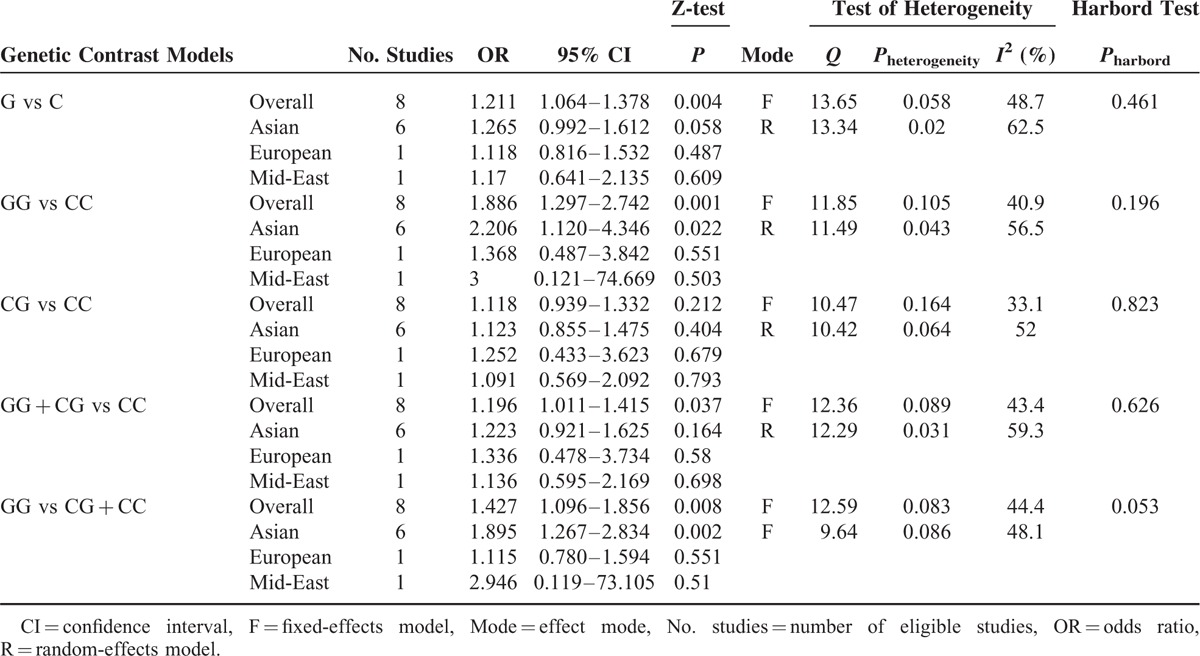
Overall and Subgroup Analysis Results of the Association Between −572 C/G Polymorphism and Hypertension

### Sensitivity Analyses

**FIGURE 3:**
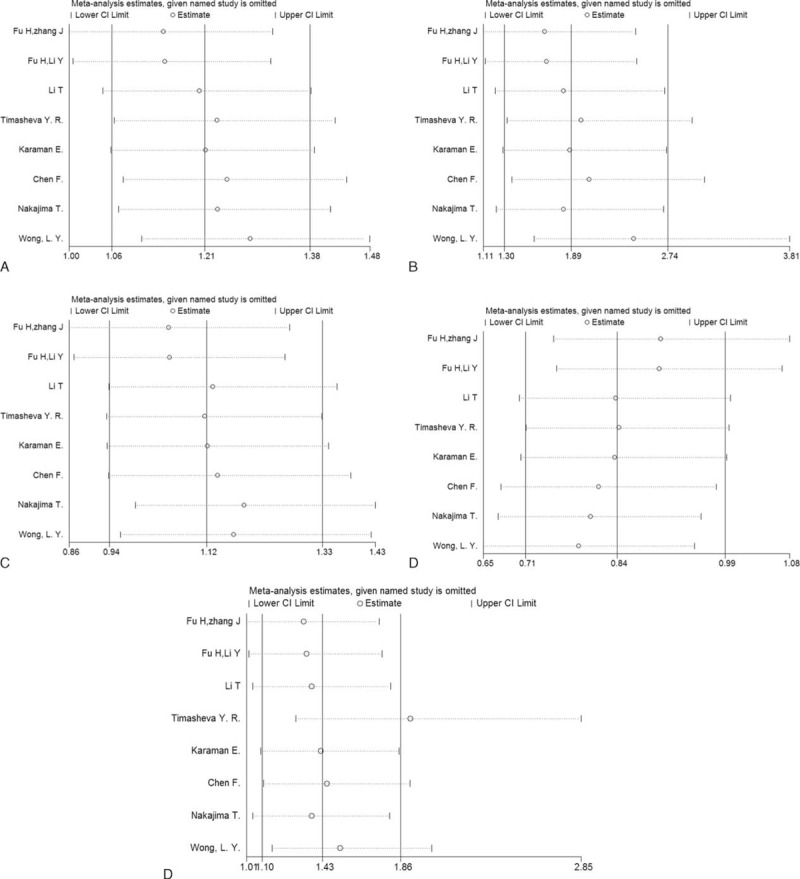
Sensitivity analysis for −572 C>G polymorphism. (A) Allelic comparison (G vs C); (B) homozygote comparison (GG vs CC); (C) heterozygote comparison (CG vs CC); (D) dominant model (GG + CG vs CC); (E) recessive model (GG vs CG + CC).CI = confidence interval, OR = odds ratio.

We conducted the sensitivity analysis to evaluate the stability of the meta-analysis (Figure 3). The results indicated that no study had a qualitative influence on the pooled ORs and 95% CIs which did not materially change in the 3 genetic models. However, the statistical significance disappeared when the studies by Fu et al^17,18^ were excluded, in turn, in the dominant model, and when the study by Fu et al^18^ was omitted from the allelic comparison. The results should be interpreted with caution.

### Publication Bias

To assess the potential publication bias, Harbord test was performed for 8 studies in the meta-analysis. The results did not show any strong statistical evidence of publication bias in all of the genetic models (Table 3) (−174 G>C).

### Statistical Analyses

**FIGURE 4:**
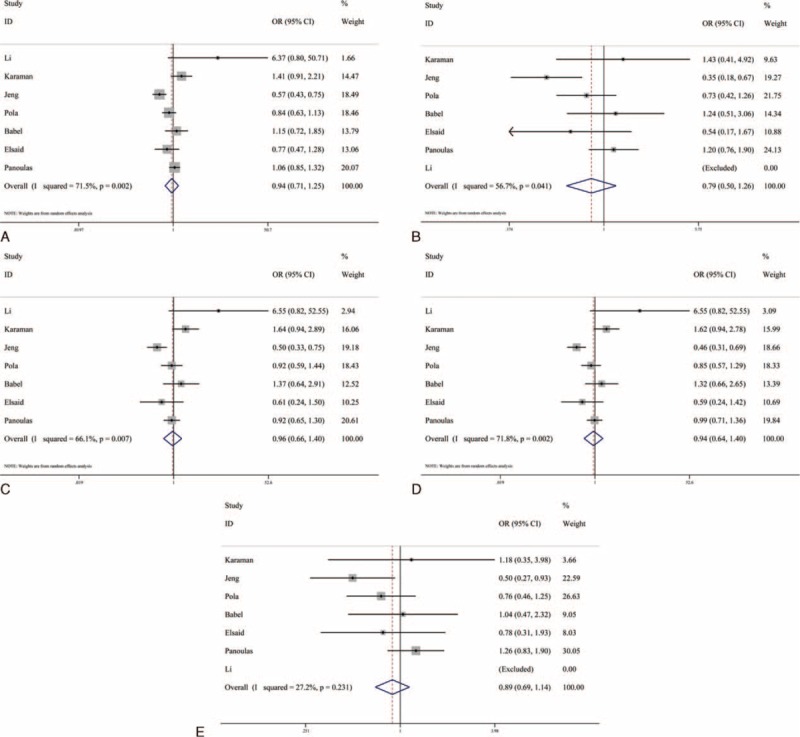
Forest plots for −174 G>C polymorphism in the overall analysis. The summary pooled ORs and 95% CIs are indicated by the white diamonds. (A) Allelic comparison (C vs G); (B) homozygote comparison (CC vs GG); (C) heterozygote comparison (GC vs GG); (D) dominant model (CC + GC vs GG); (E) recessive model (CC vs GC + GG).CI = confidence interval, OR = odds ratio.

We used the fixed-effects model and the random-effects model to merge the ORs and 95% CIs in a recessive model and 4 other models, respectively, due to the significant heterogeneity we found in the latter models (Table 4). The outcomes of the analysis showed that the frequencies of the mutant allele C in patients with hypertension were lower than those in healthy controls (C vs G: OR = 0.944, 95% CI = 0.715–1.246, *P* = 0.683; CC vs GG: OR = 0.793, 95% CI = 0.501–1.257, *P* = 0.324; GC vs GG: OR = 0.959, 95% CI = 0.655–1.402, *P* = 0.827; CC + GC vs GG: OR = 0.943, 95% CI = 0.636–1.397, *P* = 0.768; CC vs GC + GG: OR = 0.892, 95% CI = 0.695–1.144, *P* = 0.368) (Figure 4). However, the *P* values were higher than 0.05, and the 95% CIs contained 1 in all of the models, suggesting no significant associations were found between −174 G>C and hypertension. Moreover, the stratified subgroup analysis based on ethnicity was conducted and its results also indicated that there was no significance among 3 populations (Table 4). It should be noted that the study by Li et al^16^ was removed by the software because the counts of the CC genotype were 0 in both the patient group and the control group, and there was only 1 study included in the “Asian” group in the homozygote comparison and recessive model. Additionally, the *I*^2^ values, which represent heterogeneity, decreased to less than 15% in the “European” studies in all of the models and to less than 25% in the “Mid-East” studies in the homozygote comparison and the recessive model.

**TABLE 4:**
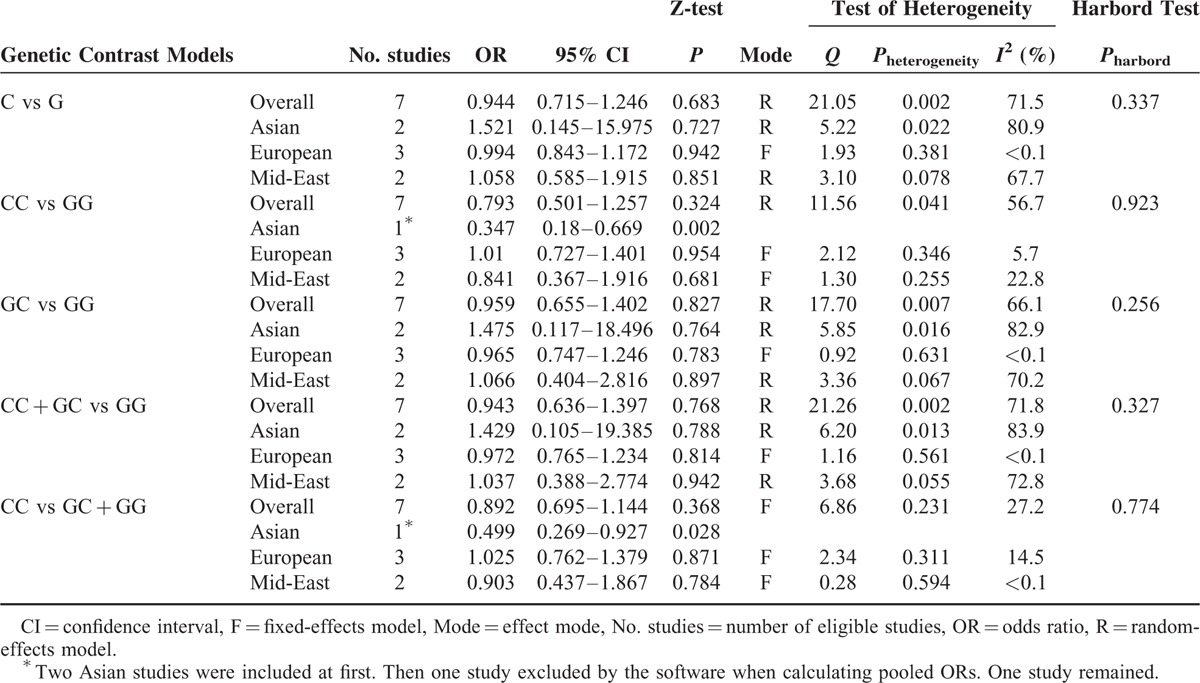
Overall and Subgroup Analysis Results of the Association Between −174 G/C Polymorphism and Hypertension

### Heterogeneity Analysis

**FIGURE 5:**
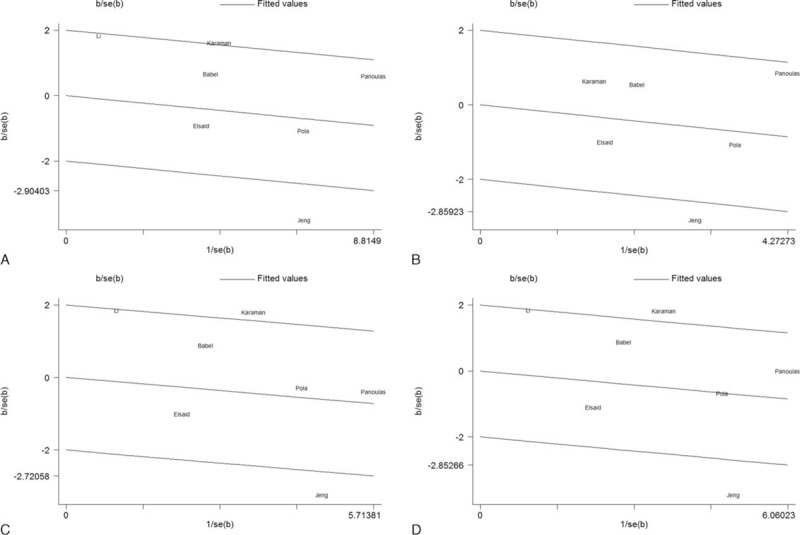
Galbraith plots for −174 G>C polymorphism in the overall analysis. The outlier dots indicate the main contributors to heterogeneity. (A) Allelic comparison (C vs G); (B) homozygote comparison (CC vs GG); (C) heterozygote comparison (GC vs GG); (D) dominant model (CC + GC vs GG). SE = standard error.

To explore the source of heterogeneity, we performed meta-regression based on genotyping methods, disease type, and sex. Unfortunately, the source of heterogeneity still remained unclear in the meta-regression, in which the results showed that the genotyping methods, the disease type, and sex were not effect modifiers (data not shown). Further exploration of the heterogeneity was conducted by Galbraith plots in 4 genetic models. We finally found that the studies by Karaman et al^74^ and Jeng et al^76^ were located outside of the 2 lines in the plots (Figure 5). After the studies by Jeng et al and Karaman et al were omitted from the corresponding models, all *I*^2^ values decreased to less than 50% in all of the genetic models, and the pooled ORs and 95% CIs were not materially altered (data not shown).

### Sensitivity Analyses

**FIGURE 6:**
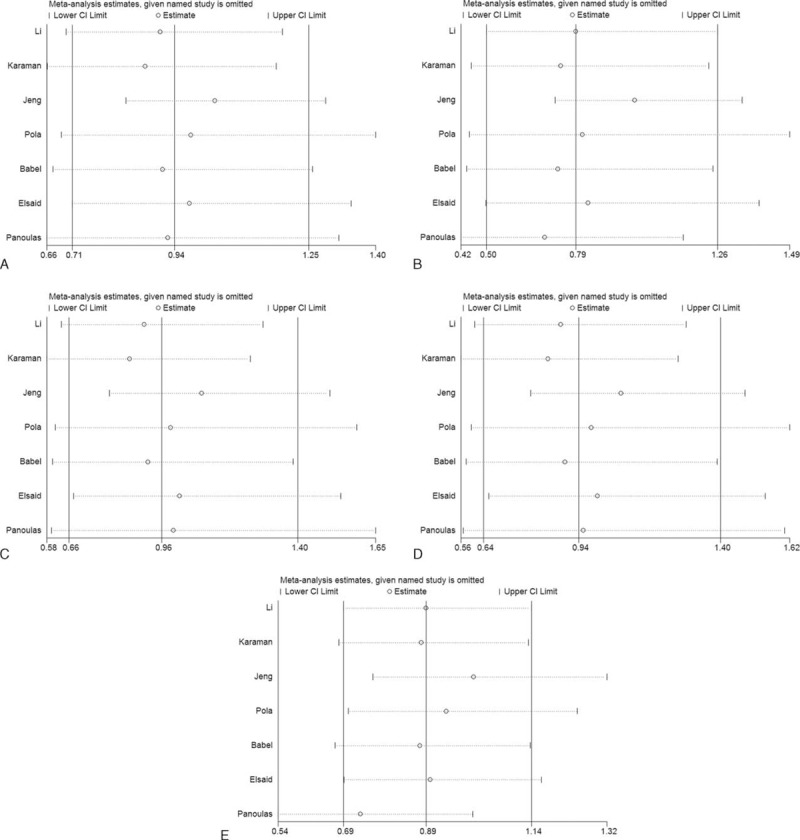
Sensitivity analysis for −174 G>C polymorphism. (A) Allelic comparison (C vs G); (B) homozygote comparison (CC vs GG); (C) heterozygote comparison (GC vs GG); (D) dominant model (CC + GC vs GG); (E) recessive model (CC vs GC + GG).CI = confidence interval, OR = odds ratio.

The sensitive analysis was carried out to determine whether the results of the meta-analysis were stable, and to confirm that there were not any studies qualitatively influencing the results and that the results were credible and reliable (Figure 6).

### Publication Bias

Finally, we tested whether a publication bias existed by Harbord method for 7 studies were included in the meta-analysis. No significant evidence supported the presence of a publication bias in all of the genetic models (Table 4).

## DISCUSSION

IL-6 does have connections with hypertension. When the cells were stimulated with lipopolysaccharide, more IL-1β and IL-6 were produced by white blood cells in hypertensive patients compared with that in the normal controls.^79^ Inflammation plays a very important role in developing atherosclerosis and cardiovascular diseases.^80^ Studies have found that inflammation may be involved in the development of hypertension and hypertension has also been described as a latent proinflammatory condition.^81^ This situation becomes a vicious spiral. IL-6 takes an active role in the final differentiation of B-cells during lymphocytes and monocytes differentiation. It is primarily secreted into serum and binds to IL-6 receptor alpha (IL-6R-α) to mediate a transcriptional inflammatory response during acute or chronic inflammation procedure. Animal experiments suggest that other cytokines, such as CRP, amyloid A, and TNF-a, are related to the occurrence of hypertension and the secretion of these cytokines is affected by IL-6 as well. Studies have already confirmed that elevated CRP level promoted the development of hypertension.^73^ Additionally, IL-6 changes the rheological characteristics of white blood cells, indirectly leading to an increase in vascular resistance.^82^ In fact, various mechanisms activated by IL-6 influence blood pressure. First, the fibrinogen synthesis system is stimulated by IL-6 to induce vessel wall collagen synthesis and it results in atherogenesis and high blood viscosity due to vascular hypofibrinolysis.^83,84^ Second, IL-6 stimulates the migration and proliferation of vascular smooth muscle cells to reconstruct vessels.^82^ Third, IL-6 regulates blood pressure by stimulating the sympathetic nervous system and controlling the expression of angiotensinogen, resulting in a high concentration of the angiotensin II and its receptors.^85^ Fourth, IL-6 increases the concentration of Ca^2+^ in vascular smooth muscle cells and causes vasoconstriction.^82^ Last, despite the direct effect on blood pressure, IL-6 is associated with obesity, coronary artery disease, diabetes mellitus,^86,87^ and catecholamine release, all of which can cooperate to promote the occurrence and development of hypertension. Thus, the progress of hypertension may be affected by anything that interferes with the copy, transcription and translation of the IL-6 gene or the secretion, migration, and proliferation of the IL-6 protein.

A large number of researchers were interested in the associations among the −572 C>G and −174 G>C variations and hypertension, but reached inclusive disquisitions. Terry et al^55^ found that the −572 C>G mutation regulated the transcription of the IL-6 gene. Malarstig et al^88^ regarded the −572 C>G mutation as a predictor of IL-6 levels above 5 ng/L in patients with cardiovascular disease, whereas a study conducted in vitro demonstrated that the −572 C>G had nothing to do with IL-6 production after lipopolysaccharide stimulation. On the contrary, patients with the G allele in the −174 G>C polymorphism displayed higher IL-6 levels compared with carriers with the C allele in the study by Burzotta et al,^89^ but conflicting results were found that the G allele and the C allele had equal effects on the patients and control populations.^90^ Although a meta-analysis performed by Niu et al^22^ maintained that the C allele was a protective factor for hypertension, only the Chinese population was included in this analysis. In addition, detailed information from the meta-analysis could not be extracted and confirmed as it was published in abstract form.

To the best of our knowledge, this is the first meta-analysis conducted to evaluate the association between the −572 C>G polymorphism and hypertension. We provided evidence that the G allele was more common in patients than in normal controls, suggesting that −572 C>G polymorphism might be a risk factor for inducing hypertension. This evidence was further strengthened by the data from the relatively conservative random-effects model. Such a result is not fully surprising, as the mutation leads to the higher expression of IL-6 in peripheral blood mononuclear cells.^91,92^ The −572 C>G mutation does not directly interfere with blood pressure, but adjusts the response of IL-6 to obesity and inflammation.^73^ In addition, serum C-reactive protein (CRP) levels, closely associated with cardiovascular disease risk, are influenced by the -572 C>G polymorphism.^93^ A subgroup analysis was conducted to discover the relationship between gene mutations and ethnicity or geographic disparities. Because only 1 European study and 1 Middle-Eastern study were enrolled in the analysis, pooled effect sizes could not be calculated, and more studies are required to evaluate the impact among these settings. The significant association disappeared in the “Asian” subgroup in the allelic comparison, the heterozygote comparison, and the dominant model, but was still positive in the remaining models. We suspected that the random-effects model led to the relatively conservative result. In addition, each subgroup in the analysis had relatively smaller sample sizes, which caused an inevitable decrease of the test power, potentially resulting in false negative. Additionally, in 2 genetic models, the results of the sensitivity analysis revealed the instability that was probably due to the small number of eligible studies. Additionally, after examining the study participants in the “Materials and Methods” section of the 2 articles that caused the instability,^17,18^ we found that neither of them clarified whether the patient groups included those patients whose blood pressure was normal when the study was carried out, but used to have a history of hypertension before. This approach might become a source of heterogeneity that could alter the results. For these reasons, the results of the population stratification should be interpreted with caution. More studies are required to consolidate the validity.

As the −572 C>G polymorphism was found at a prevalence of 75% in eastern Asian populations,^94^ our result may contribute to the public health management and pathogenesis research of hypertension, especially in Asian area. Further studies with larger sample sizes are needed to determine whether routine screening for the −572 C>G polymorphism is warranted and recommended in clinical practice as a precaution for hypertension at early disease stage.

As for −174 G>C polymorphism, we did not find any significant associations in all of the genetic models based on a random-effects model. Because previous reports showed that the −174 G>C polymorphism varied in different geographical regions and ethnic groups,^14,55^ a stratified analysis was carried out based on ethnicity. We failed to detect any significant differences among ethnicities. Intuitively, our results were inconsistent with the previous meta-analysis by Niu et al^22^ because our meta-analysis was superior due to the following reasons: first, we included more recently published studies,^11,16,74,75^ which were not available at the time of the previous meta-analysis. Second, the −174 G>C polymorphism was rare in eastern Asian populations^94^ and had a relatively high allele frequency in Caucasians (40%).^95^ Physiologically, important contributors of cardiovascular diseases, such as CRP, seemed to be unaffected by the −174 G>C mutation.^96^ Nonetheless our results and analysis can provide precise information about a wider geographical scope.

In addition to providing a convincing conclusion, the meta-analysis aims to determine the source of heterogeneity. Taking the existence of heterogeneity into consideration in the analysis of the −174 G>C polymorphism, we conducted a subgroup analysis and only found a decrease in the *I*^2^ among the “European” studies. A meta-regression was further implemented to explore the underlying factors. Unfortunately, the source of controls, sex, disease type, and genotype methods did not prove to be effect modifiers. In the end, Galbraith plots were drawn to detect the outliners leading to heterogeneity. All *I*^2^ values immediately decreased to below 50% and the *P* values of the Q-test were greater than 0.1 after excluding the Jeng et al^76^ or Karaman et al^74^ study. Moreover, the 95% CIs still contained 1 in 4 genetic models after removing these studies, proving that our results were robust. The results of the sensitivity analysis also supported this view.

High genotyping accuracy was the basis of quality control and ensured the precision and objectivity of the conclusion. In this meta-analysis, more than half of the studies used PCR-RFLP, which is considered a classic genotyping method. The meta-regression also demonstrated that the genotyping method was not an influential factor. Some studies even performed 2 more genotyping methods to verify the results (eg, the MassARRAY system). All studies provided detailed and explicit information about primer sequences, methods for DNA extraction, and the PCR reaction programs. In a word, our conclusion is reliable.

Some certain limitations of this meta-analysis should be noted. First, there were only 1 European study and 1 Mid-East study in the analysis of the −572 C>G polymorphism, and 1 Asian study in the recessive model and homozygote comparison for the −174 G>C mutation (Li et al was excluded by the software when merging the ORs), which prevented merging ORs and 95% CIs in the subgroup analysis. As a result, we had lower statistical power that limited the extrapolation range in the subgroups. Further, some confounding factors might influence the subgroup analysis. The studies had interaction effects with each other. For example, different geographical areas had not only different ethnicities, but also different dietary habits that could cause hypertension. It was difficult to completely offset these factors due to the original study designs. Thus, the results of the stratified analysis should be interpreted with caution. Second, we could not exhaustively describe the effect of gene-to-gene and gene-to-environment factors because of the insufficient data. Inheritance factors are thought to be responsible for only about 30% to 60% of the rise in blood pressure,^97^ and no single gene played a major role at the molecular level. Future studies are required to estimate the effects. Third, some relevant studies could not be included due to insufficient raw data, improper study designs, or unsuitable publication formats, such as abstracts. This led to the small number of included articles which caused instability when we analyzed the −572 C>G polymorphism. Although the results of Harbord method showed that there was no evidence of publication bias in this meta-analysis, the possibility of bias cannot be completely rejected. More evidence is required and future meta-analyses should include more studies.

In conclusion, our meta-analysis demonstrated that the −572 C>G polymorphism is correlated with the development of hypertension in Asian population. More studies, especially those conducted with other races, are required to strengthen our conclusion and to explore the relationship between the −572 C>G and hypertension globally. In contrast, the −174 G>C polymorphism did not have a significant association with hypertension. Further large-scale studies are needed in the future, particularly for the consideration of gene-to-gene and gene-to-environment interactions.

## Supplementary Material

Supplemental Digital Content
